# Gill chamber and gut microbial communities of the hydrothermal shrimp *Rimicaris chacei* Williams and Rona 1986: A possible symbiosis

**DOI:** 10.1371/journal.pone.0206084

**Published:** 2018-11-02

**Authors:** Vincent Apremont, Marie-Anne Cambon-Bonavita, Valérie Cueff-Gauchard, David François, Florence Pradillon, Laure Corbari, Magali Zbinden

**Affiliations:** 1 IFREMER, Univ Brest, CNRS, Laboratoire de Microbiologie des Environnements Extrêmes, Plouzané, France; 2 Unité Biologie des ORganismes et Ecosystèmes Aquatiques (BOREA), Sorbonne Université, MNHN, CNRS, IRD; Equipe Adaptation aux Milieux Extrêmes (AMEX), 7 Quai St Bernard, Paris, France; 3 Ifremer centre Bretagne, ZI de la Pointe du Diable, Laboratoire Environnement Profond, REM/EEP/LEP, Plouzané, France; 4 Muséum National d’Histoire naturelle, Institut de Systématique, Évolution, Biodiversité ISYEB—UMR 7205 –CNRS, MNHN, UPMC, EPHE, Paris, France; Academia Sinica, TAIWAN

## Abstract

*Rimicaris chacei* Williams and Rona 1986, formerly named as *Chorocaris chacei*, is a caridean shrimp living in deep-sea hydrothermal ecosystems. This shrimp is endemic to the Mid Atlantic Ridge (MAR) and lives at the periphery of aggregates of its well-known congeneric *R*. *exoculata* Williams and Rona 1986. Contrasting with the very dense and mobile clusters formed by *R*. *exoculata*, *R*. *chacei* lives in small groups of several individuals that are not very mobile. Although devoid of the characteristic hypertrophied cephalothorax of *R*. *exoculata*, which harbors the ectosymbionts, a microbial community has also been reported in the cephalothorax of *R*. *chacei*. Previous data on morphology, behavior and isotopic values indicate a diet based on a combination of feeding on its epibiotic bacteria and scavenging or occasional predation. In this study, our objective was to describe, for the first time, the distribution, morphology and phylogeny of the microbial communities associated with *R*. *chacei*. This species is significantly less studied than *R*. *exoculata*, but nevertheless represents the only other known example of symbiosis in crustaceans of MAR hydrothermal vent sites. Microbial communities have been observed at the same locations as in *R*. *exoculata* (mouthparts, branchiostegites and digestive tract). However, in *R*. *chacei*, the surfaces occupied by the bacteria are smaller. The main lineages are affiliated to *Epsilon* and *Gammaproteobacteria* in the cephalothorax and to Deferribacteres, Mollicutes, *Epsilon* and *Gammaproteobacteria* in the digestive tract. Comparison with the well-described bacterial communities of *R*. *exoculata* and hypotheses about the role of these communities in *R*. *chacei* are discussed.

## Introduction

Deep abyssal areas are characterized by their very low biomass and highly diverse communities [1,2], but deep-sea hydrothermal vent ecosystems are endemism hotspots harboring high biomasses and low diversity. The highest biomass species encountered around the vent emissions (for review see [3]) rely on trophic symbioses, based on chemosynthetic microorganisms [4]. These relationships were initially and most commonly described as endosymbioses (e.g., *Riftia pachyptila* Jones 1981 or *Bathymodiolus* mussels, see review in [3]). Among crustaceans, symbioses have only been reported in a few species and as ectosymbioses (e.g., in some galatheid crabs, barnacles and shrimps, see [3] and [5] for review). The best-studied example is the shrimp *Rimicaris exoculata* from the Mid Atlantic Ridge (MAR), which harbors a double symbiosis. A first bacterial community is located in the gut [6] with long thin (0.2 μm x 15 μm) single-celled bacteria inserted between the microvilli of the midgut epithelium [6], and rods and cocci within the gut content [7]. This bacterial community is composed of four major groups: Deferribacteres, Mollicutes, *Epsilonproteobacteria*, and, to a lesser extent, *Gammaproteobacteria* [6, 7, 8]. The role of these lineages is still poorly understood, as they do not seem to be highly implicated in the host’s nutrition, in contrast to the second bacterial community located in the cephalothorax [9]. There, bacteria are mostly present on the bacteriophore setae of the hypertrophied exopodites of the second maxilla (scaphognathites) and first maxillipeds, and on the internal tegument of the carapace (branchiostegites) [10, 11]. This community is composed of rod, coccoid, thick and thin filamentous bacteria [11, 12]. A great phylogenetic diversity has been found within this community, with bacteria belonging mostly to the Proteobacteria phylum, including two major groups: *Epsilon*- and *Gammaproteobacteria*, and lower abundance of *Alpha-*, *Beta*-, *Delta*-, and *Zetaproteobacteria* [13–17]. Sulfide, methane, iron and hydrogen oxidation were proposed and / or demonstrated as major chemoautotrophic metabolisms within the epibiosis [9, 12, 13, 15, 18]. The supply of organic carbon to the host by cephalothoracic bacteria was strongly suspected by isotopic analyses [19, 20]. Ponsard *et al*. [9] demonstrated that this supply takes place in the form of a transfer of soluble bacterial byproducts across the gill chamber integument by transtegumental absorption.

The *Rimicaris* genus now comprises nine species. It has recently been revised by invalidating the genus *Chorocaris* and transferring the species of this genus into *Rimicaris* [21]. Within the *Rimicaris* genus, two other species (*R*. *kairei* Watabe and Hashimoto 2002 and *R*. *hybisae* Nye, Copley and Plouviez 2011) present a hypertrophied cephalothorax but, to date, associated bacteria have only been described from the scaphognathites of *R*. *hybisae* [22, 23]. Among species previously assigned to the genus *Chorocaris*, none have been described with an enlarged cephalothorax and *R*. *chacei* is the only one in which the presence of epibiotic bacteria has been reported [10]. *R*. *chacei* has probably not received as much attention as its congeneric *R*. *exoculata* because it is much less abundant. This species lives along the MAR, in small communities (up to 50 ind m^-2^) in the periphery of *R*. *exoculata* aggregates (containing up to 3000 ind m^-2^) [24, 25]. Although *R*. *chacei* scaphognathites and exopodites are not hypertrophied, they nevertheless bear plumose setae on both sides, yet less numerous than in *R*. *exoculata* [10]. Dense filamentous microbial communities have been observed on mouthparts and inside the carapace, but the areas colonized are smaller than in *R*. *exoculata* [10, 11]. The occurrence of bacteria in the digestive tract has not yet been investigated in *R*. *chacei*. Stable isotope analyses show that in small adults of *R*. *chacei* much of the carbon is derived from chemosynthetic bacteria but that in larger individuals scavenging or occasional predation on *R*. *exoculata* or mussels may occur, although there is still a substantial input from epibiotic bacteria [26].

In the present study, we provide a detailed description and characterization of the association between *R*. *chacei* and its microbial communities (in both the cephalothorax and the digestive system) and we draw hypotheses about their roles in comparison with those found in *R*. *exoculata* symbiosis. The differences and points in common between the symbioses of *R*. *chacei* and *R*. *exoculata* are discussed.

## Material and methods

### Sampling

Specimens of *R*. *chacei* were collected at several hydrothermal vents along the MAR: Lucky Strike (37°17’N; 32°16.3’W; 1730 m depth) and Rainbow (36°13’N; 33°54’W; 2350 m depth) during the MoMARdream-Naut (July 2007) and BioBaz cruises (August 2013); and TAG (26°08’N; 44°49.6’W; 3700 m depth) and Snake Pit (23°23’N; 44°56.1’W; 3480 m depth) during the BICOSE cruise (January 2014). Shrimps were collected using the suction sampler of the Human operated vehicle "Nautile" and the Remotely Operated Vehicle "Victor 6000" from the research vessel "*Pourquoi pas*?". Once on board, individuals were dissected for tissues of interest (branchiostegites (LB), scaphognathites (Sc) and digestive tract: foregut (FG) and midgut (MG)) (Table 1). For molecular studies, these tissues were frozen (-80°C) on board and DNA extractions were performed in the laboratory back on shore. For TEM and SEM, samples were fixed in a 2.5% glutaraldehyde─seawater solution and later post-fixed in osmium tetroxide. For fluorescent *in situ* hybridization (FISH), samples were fixed for 2 h in a 3% formaldehyde—sterile seawater solution and further treated as described in [6]. In order to observe well-developed bacterial communities in the cephalothorax, all specimens analyzed in this study were chosen to be of comparable size and at the preecdysial stage (i.e., just before a molt), which was identified by branchiostegite coloration, as described for *R*. *exoculata* [27]. Number of samples studied per site and for each experiment are reported in Table 1.

**Table 1:** Number of specimens used per site and for each experiment in this study.

	TEM			SEM			16S				FISH				N
	LB	SC	MG	LB	SC	MG	LB	SC	FG	MG	LB	SC	FG	MG	
Lucky Strike	1	1	1	1	1	1	1	1	1	1					2
Rainbow	1	1	1	1	1	1	3	3	3	2	2	2		1	5
TAG	1	1	1	1	1	1	1	1	1	1	1	1		1	4
Snake Pit	1	1	1	1	1	1	1	1	1	1	1	1		1	4

LB = Branchiostegites, SC = Scaphognathites, FG = Foregut, MG = Midgut. N = total number of individual per site

Lucky Strike site is part of Portugal's EEZ and a "Protected Marine Area" (OSPAR). Work authorization in the waters of the Portuguese EEZ is issued by the Portuguese authorities. No specific permissions were required to collect samples in international deep-sea waters (Rainbow, TAG and Snake Pit sites). The study did not involve endangered or protected species.

### DNA extraction and PCR amplification

DNA from all tissues (LB, SC, FG and MG) was extracted and purified using the Nucleospin soil kit (Macherey-Nagel) following the manufacturer's recommendations. Bacterial 16S rRNA gene fragments were PCR-amplified in 30 cycles at an annealing temperature of 48°C, using the general bacterial primer set E8F (5’ AGA GTT TGA TCA TGG CTC AG 3’) and U1492R (5’ GTT ACC TTG TTA CGA CTT 3’) [28]. They were then purified with a Nucleospin Extract II kit (Macherey-Nagel) following the manufacturer's recommendations.

### Cloning and sequencing

The amplified PCR products were cloned using the TOPO TA cloning kit (Invitrogen) following the manufacturer’s instructions. The plasmid inserts were checked by amplification using M13F and M13R primers. Positive clones were then sequenced at GATC Biotech (Germany) on a Sanger ABI 3730xl DNA Sequencer.

### Phylogenetic and communities analysis

Sequences (16S rDNA) were checked for quality: length (sequences too short compared with literature data were removed), HQ % (according to MOTHUR use, sequences with HQ% below 35% were removed), and number of repeated nucleotides in sequences under 6. They were then sorted into “Forward” and “Reverse” sequences based on PCR primer identification (E8F and U1492R respectively) and checked for chimeras using UCHIME [29] and Decipher [30] algorithms. Sequences were aligned using the MAFFT [31] algorithm and refined manually with Geneious 8.1.8 [32]. All phylogenetic trees were built with Geneious 8.1.8. Phylogenetic analyses were performed on the basis of evolutionary distance (Neighbor Joining; [33]) using the general time reversibility (GTR) model for the correction matrix. The robustness of phylogenetic reconstructions was tested by bootstrap re-sampling (x1000) [34]. Sequences showing more than 97% similarity using the Furthest method [35] with MOTHUR software [36] were considered to be sufficiently related and were grouped in the same phylotype (OTU: Operational Taxonomy Unit). Rarefaction curves (see S2 and S3 Figs) were created to estimate sequencing depth. The Bray–Curtis calculator [37] was used to generate a β diversity matrix. Singletons were deleted from the data to generate phylogenetic trees but are present in the diversity data.

The rarefaction curves, Simpson indices and related evenness of Simpson index were obtained with MOTHUR (at 97% similarity) for all libraries [35, 36].

Good’s coverage was calculated as a percentage according to the following relation:

C = [1-(n/N)] x 100, where n represents the number of phylotypes appearing only once in a library and N being the library size [38].

### Fluorescent in situ hybridization (FISH)

The FISH protocol used was described previously by Durand *et al*. [6]. Briefly, 3%-formaldehyde fixed dissected tissues were embedded in polyethylene glycol distearate-1-hexadecanol (9: 1) resin (Sigma, St. Louis, MO). Resin blocks were then cut into 6–10 μm sections using an RM 2165 microtome (Reichert-Jung, Germany). Sections were hybridized using several published probes (Table 2). The probe sequences have been compared to our sequences to check their specificity and determine their mismatches. The hybridization temperature was the same for all samples treated (46°C). Observations and imaging were performed using an ApoTome Axio Imager Z with a COLIBRI system (Zeiss, Jena, Germany) using ZEN software (Zeiss, Jena, Germany).

**Table 2:** Fluorescent probes used in this study.

Specificity	Probe name	Sequence (5’-3’)	Fluorescent dye	% Formamide	References
Archaea	Arch915	GTGCTCCCCCGCCAATTCCT	Cy3	10–20–30–40	[67]
Eubacteria	Eub338	GCTGCCTCCCGTAGGAGT	Cy3 or Cy5	10–20–30–40	[68]
Gamma proteobacteria	GAM42a	GCCTTCCCACATCGTTT	Cy3 or Cy5	10–20–30–40	[69]
Epsilon proteobacteria	EPSY549	CAGTGATTCCGAGTAACG	Cy3 or Cy5	10–20–30–40	[70]

### Scanning electron microscopy (SEM)

Before preparation, samples were observed under a stereomicroscope and photographed (Olympus SZX12 equipped with an Olympus U-CMAD3 camera). Samples were dehydrated through an ethanol series and critical point dried (Emitech K850). Samples were then gold-coated with a JEOL JFC-1200 fine coater. Observations were performed with a scanning electron microscope (Hitachi SU3500), operating between 5 and 25kV according to the sample.

### X-ray microanalysis

Elemental energy-dispersive X-ray microanalyses (EDX) were performed on the samples used for SEM. X-ray microanalyses and elemental mappings were carried out using a scanning electron microscope (FEI Quanta 200) operating at 20 kV, and acquired with an energy dispersive X-ray detection system (SDD X-Max 80 mm detector).

### Light microscopy (LM) and transmission electron microscopy (TEM)

Sections were prepared as described in Zbinden *et al*. [13]. Semi-thin sections were observed by light microscopy (using a Zeiss microscope, Jena, Germany). Ultra-thin sections were stained with Urany-less and lead citrate (Delta Microscopies) and observed on a Zeiss 912 transmission electron microscope operating at 80 kV.

### Nucleotide sequence accession numbers

The sequences from this study are available through GenBank under the following accession numbers: LT855310 to LT855375 (16S rRNA genes sequences).

## Results

### Shrimp morphological observations

**Fig 1:**
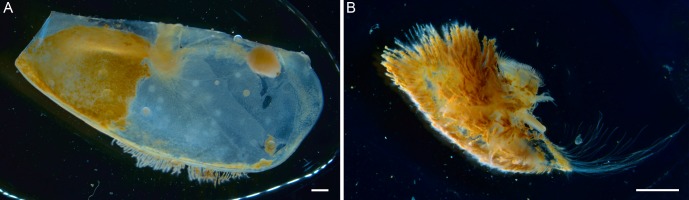
Stereomicroscopic view of the tissues bearing the symbionts in *R*. *chacei*. A) Inner face of a branchiostegite and B) Scaphognathite, showing also the occurrence of mineral deposits. Scale bars: A, B = 1.5 cm.

Light microscopy observations of the inner branchiostegites revealed two distinct areas (Fig 1A): (i) the anterior part (corresponding to one third of the branchiostegite surface) showed an orange coloration, due to mineral deposits; and (ii) the posterior part facing the gills, which was translucent. The scaphognathites (Fig 1B), which bear long bacteriophore setae on both sides, were also covered in orange deposits. Stomach and gut of shrimps from the Rainbow site were full of orange/brown content, whereas those from Snake Pit and Lucky Strike were rather full of black/grey content, those from TAG having both orange and black particles in their content (S1 Fig).

### Morphology and distribution of bacteria

**Fig 2:**
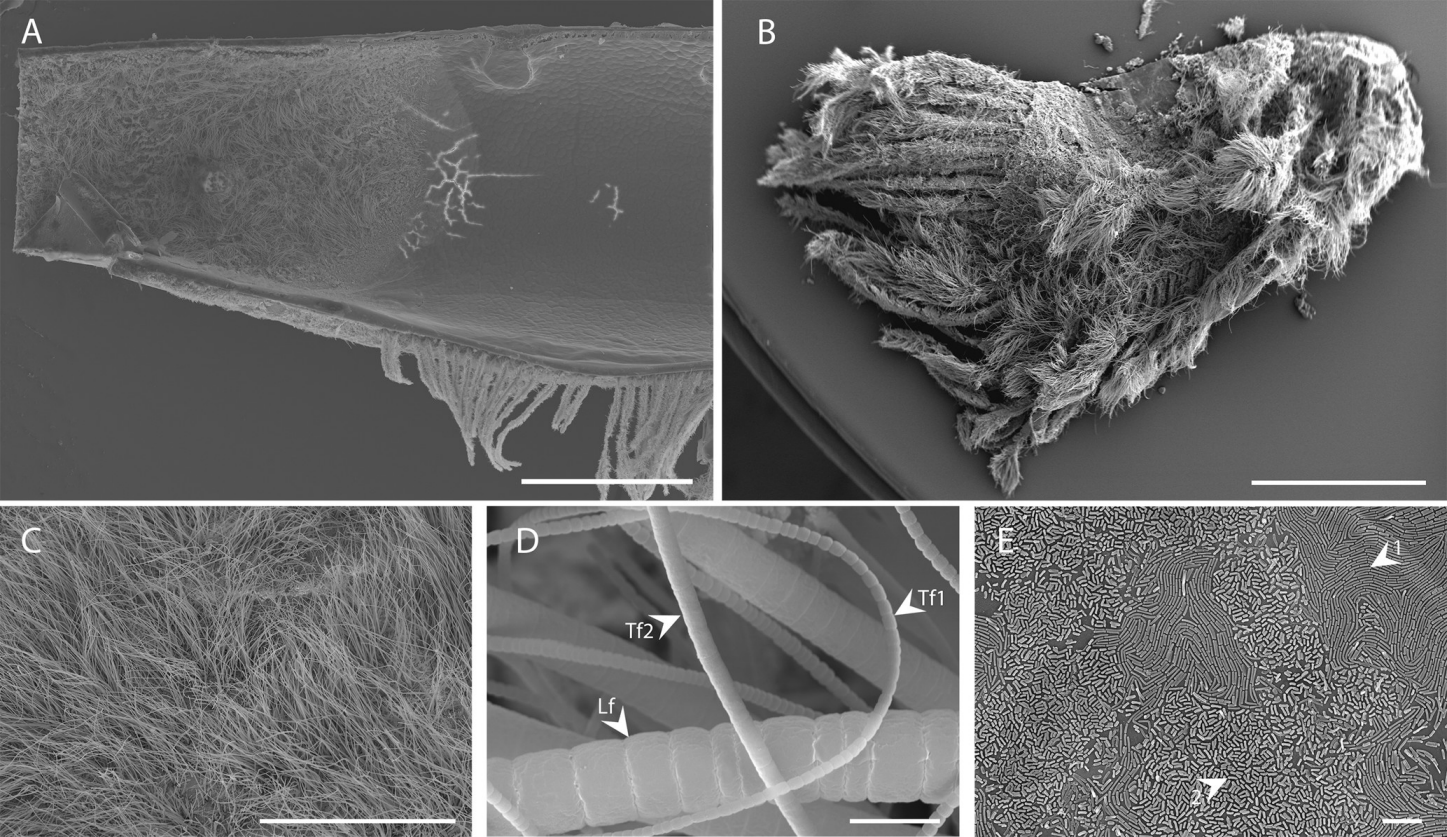
SEM images of the bacterial colonization in the cephalothorax of *Rimicaris chacei*. A) Overview of the inner side of the branchiostegite showing colonized (on the left) and uncolonized (on the right) areas. B) Overview of the dorsal side of the scaphognathite. C) Enlargement of the filamentous bacterial mat of the inner side of the branchiostegite. D) High magnification of the three filamentous morphotypes: large filaments (Lf), thin filaments of type 1 (Tf1) and thin filaments of type 2 (Tf2). E) The two bacillus morphotypes (types 1 and 2) on the inner side of the branchiostegite. Scale bars A, B = 1 mm, C = 400 μm, D, E = 5 μm.

**Fig 3:**
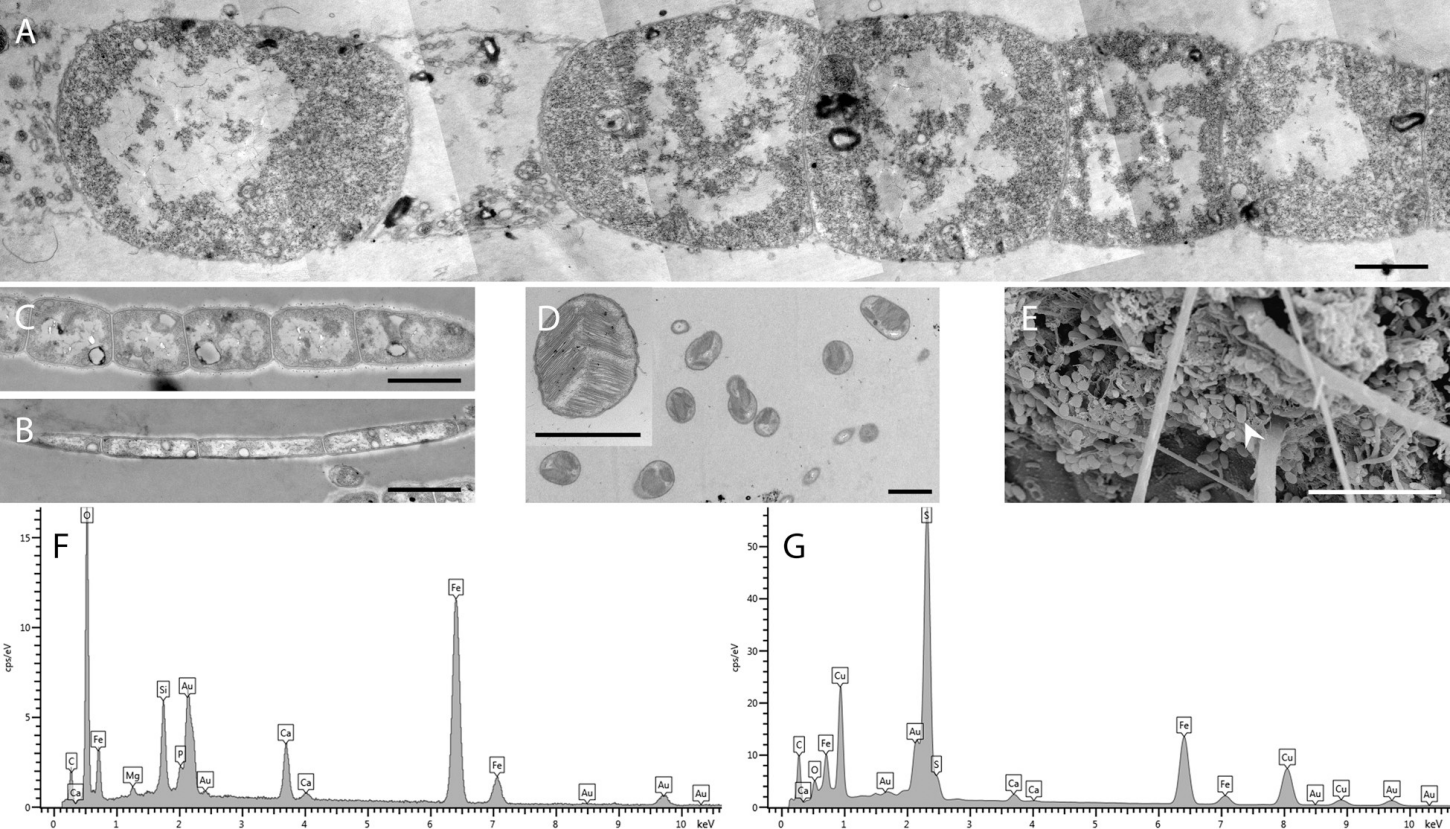
TEM and SEM observations of the different bacterial morphotypes and X-ray analysis of the associated minerals. A) TEM view of a large filament. B) TEM view of a type 1 thin filament. C) TEM view of a type 2 thin filament. D) TEM view of methanotrophic-like bacteria. E) SEM image of a methanotrophic-like bacteria morphotype (arrowheads). (F-G) Elemental X-ray microanalysis of mineral deposits on branchiostegite of a specimen from the Rainbow site. Most of this rusty-colored mineral deposit is composed of iron and oxygen, supposedly as iron oxide (F). Fe/Cu sulfide particles can be occasionally observed among the oxide particles (G). Scale bars A, B, C, D and insert in D = 1 μm, E = 10 μm.

SEM observations of the branchiostegites (Fig 2A and 2C) and scaphognathites (Fig 2B) showed a dense microbial colonization. Combining SEM and TEM, a total of six bacterial morphotypes were observed: three type of filaments (Figs 2D, 3A and 3C), two rod-shaped (Fig 2E), and one coccobacillus morphotype (Fig 3D and 3E). The sizes and abundances of bacteria on specimens from each site are summarized in Table 3. Filamentous morphotypes comprised a large type (Lf, Figs 2D and 3A) and two thin types: one with short and thin cells (type 1, Tf1, Figs 2D and 3B) and one with longer and larger cells (type 2, Tf2, Figs 2D and 3C). Among the rods, the first type was longer and thinner than the second type (Fig 2E). The coccobacillus morphotype (Fig 3E) presented stacks of internal membranes characteristic of methanotrophic-like bacteria type I (Fig 3D) [39]. These occurred in small aggregates of four to seven bacteria. All these morphotypes were present on the branchiostegites and scaphognathites of the shrimp from each studied vent site, except for large filamentous morphotypes, which were not observed on TAG specimen, and methanotrophic-like bacteria, which were not observed on TAG or Snake Pit specimens.

**Table 3:** Cell sizes of the various bacterial morphotypes observed and they relative abundance for each hydrothermal vent site.

Morphotype	Diameter	Height	Lucky Strike	Rainbow	TAG	Snake Pit
Large filament	2,42 ± 0,58 (n = 81)	2,57 ± 0,75 (n = 57)	+++	+++	-	+++
Thin filament (type 1)	0,80 ± 0,07 (n = 114)	0,70 ± 0,12 (n = 109)	+++	+++	+++	+++
Thin filament (type 2)	1,12 ± 0,13 (n = 27)	5,61 ± 1,07 (n = 43)	++	++	++	++
Thick rods (type 1)	0,26 ± 0,04 (n = 89)	1,6 ± 0,33 (n = 108)	+++	+++	+++	+++
Thin rods (type 2)	0,28 ± 0,06 (n = 70)	0,94 ± 0,17 (n = 70)	+++	+++	+++	+++
Coccobacilli	0,66 ± 0,09 (n = 91)	1,07 ± 0,17 (n = 124)	+++	+++	-	-

-: absent, ++: abundant, +++ very abundant. Values are given in μm ± standard deviation.

In all SEM observations (n = 4 individuals, LB and SC for each), mineral deposits were always located on the bacterial communities and nowhere else. Qualitative analyses with X-ray microanalysis revealed two major types of mineral deposit. The first, and largely dominant, mineral was mainly composed of iron (major peaks Kα at 6.400 keV and Kβ at 7.059 keV) and oxygen, probably as iron oxides (presented here for the Rainbow site, Fig 3F). The second type occurred only rarely and was mainly composed of sulfur (major Kα peak at 2.307 keV) and iron, but occurred together with Cu (Kα at 8.041 keV and Kβ at 8.907 keV), probably as iron/copper sulfides (Fig 3G from the Rainbow site). The same profiles were obtained for the minerals associated with specimens from the other vent sites.

**Fig 4:**
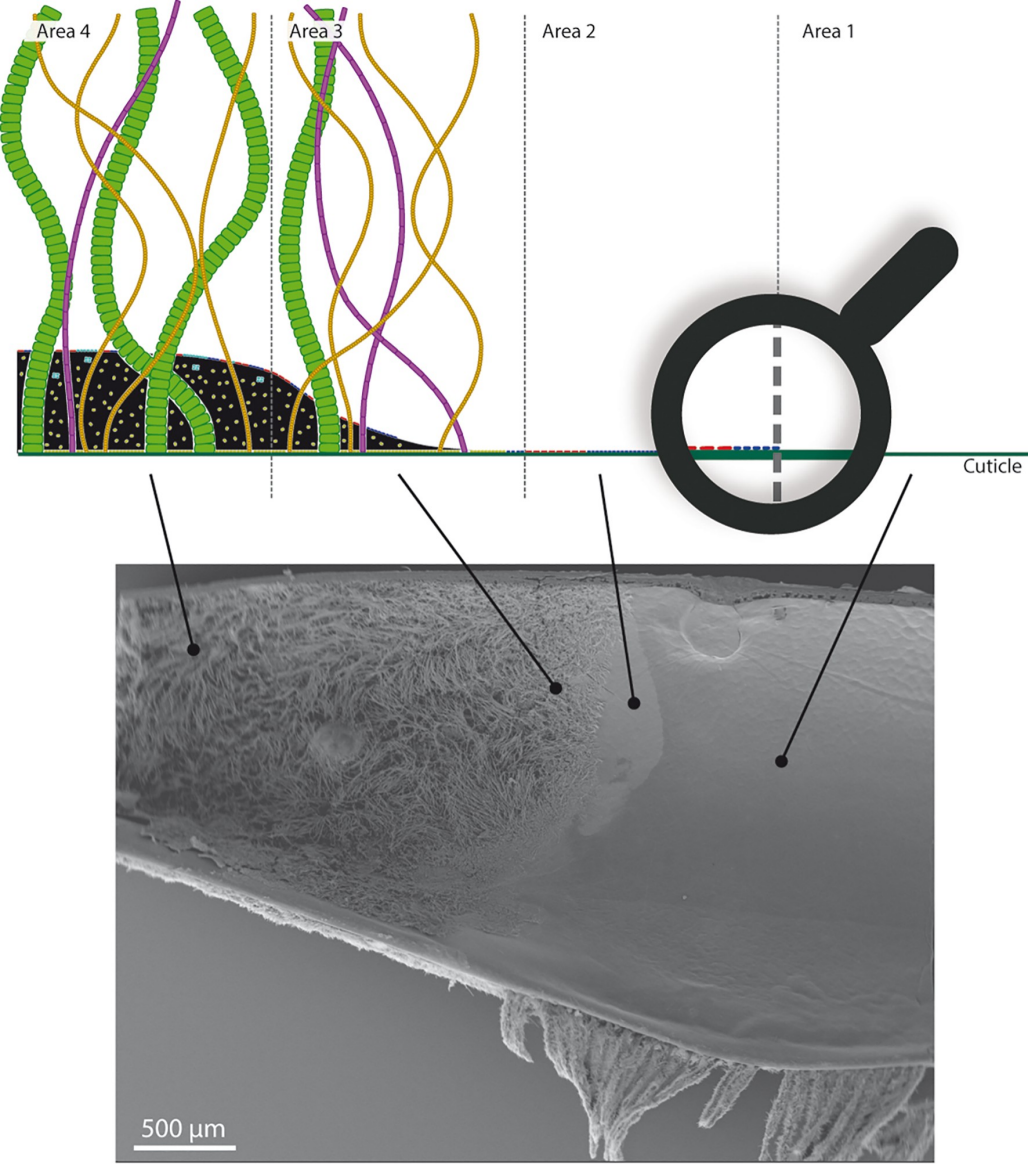
Bacterial colonization area on the inner side of the branchiostegite. A) Schematic representation of the four bacterial colonization areas (with accurate relative sizes of the different bacterial morphotypes). The green line under the drawing is the shrimp cuticle. Type 1 rods are colored in red, type 2 rods in blue (on the cuticle on area 2, and on the mineral deposits on area 3 and 4), large filaments in green, type 1 thin filaments in orange, type 2 thin filaments in purple and methanotrophic-like bacteria in turquoise (within and abose the mineral deposits). B) SEM micrograph showing the inner side of a branchiostegite and the four areas described in the text and schematized above.

Combining all our observations, we identified four distinct bacterial colonization areas on branchiostegites, as summarized in Fig 4A. Area 1 was characterized by a total absence of bacteria and mineral deposits. Area 2 was characterized by the occurrence of a monolayer of the two rod morphotypes (type 1 colored in red and type 2 in blue on Fig 4A), and no associated mineral deposits. Areas 1 and 2 corresponded to the translucent part of the host branchiostegite described above by macroscopic observation (Fig 1C). Area 3 was characterized by a thick bacterial mat composed of the three filamentous morphotypes (Lf colored in green, Tf1 in orange and Tf2 in purple on Fig 4A), as well as an increasing amount of mineral deposits towards the fourth area. Above, inside and under this deposit, rods of the two types were also present. Area 4 was similar to area 3, but with a greater thickness of mineral deposits. It was also the only area with methanotrophic-like bacteria (Fig 3D, colored in turquoise on Fig 4A). Areas 3 and 4 corresponded to the anterior part described above by macroscopic observation.

Scaphognathites were usually heavily mineralized (Fig 1D), showing occurrence of orange-brown deposits on both sides, as well as on the bacteriophore setae, along with the different morphotypes of rods and filaments (Fig 2B).

**Fig 5:**
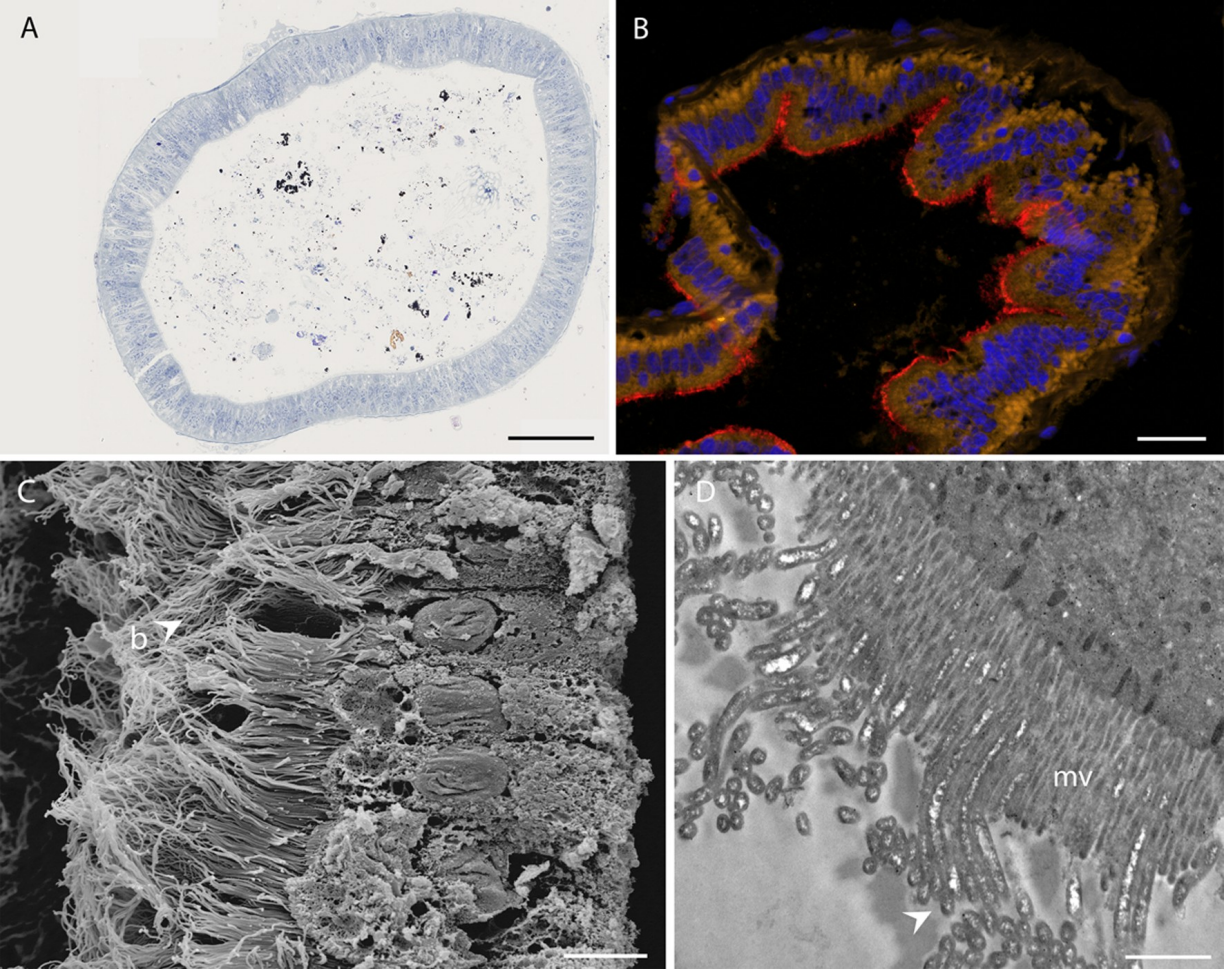
*Rimicaris chacei* midgut. A) Photonic observation of a semi-thin section of the digestive tract (hindgut), showing black and brown mineral particles, as well as organic matter stained with toluidine blue. B) FISH of a midgut transversal section stained with DAPI (blue), and hybridized with the Eubacteria general probe Eub338-Cy5 (red) and autofluorescence of intestinal cells (yellow). C) SEM image of a bacterial mat (b) on intestinal wall cells. D) TEM image of filamentous bacteria (arrows) inserted between microvilli (mv) of intestinal cells. Scale bars: A = 10 μm, B = 1 μm, C = 50 μm, D = 10 μm.

Cross-sections of *R*. *chacei* midgut (Fig 5A) revealed that the gut content is mainly composed of black and brown minerals, probably as iron sulfides and oxides, along with organic matter in the form of a few cuticle fragments and ingested bacteria (filaments and coccoid cells). Epithelial gut cells were densely colonized by a single morphotype of long (up to 20–25 μm) and thin (about 0.18 ± 0.02 μm; n = 100), unsegmented filamentous bacteria (Fig 5B and 5C), inserted between the microvilli (Fig 5D). These filamentous bacteria were observed in dense communities within the gut of all specimens, with no visible difference in their distribution and abundance.

### Diversity of *Rimicaris chacei* bacterial communities

Due to environmental and technical limitations, our results are based on only a few samples per site. PCR and cloning approaches are somewhat limited by amplification quality and the number of clones treated. Considering all these possible sources of bias, all samples were treated in the same way throughout the study, combining multiple approaches (microscopy, FISH and molecular analyses), as in previous studies [13, 14, 16], which enable us to make some comparisons between the different specimens and sites [40].

Good’s coverages were inferior to those of previous studies on *R*. *exoculata* due to slight differences in technical analysis. In [16] and [6], OTUs (threshold at 97%) were manually inferred from phylogenetic tree analyses. Here we used methods specially developed for clustering to limit possible phylogenetic tree and manual assignment of OTUs bias, although using NJ tree reconstruction for comparison with previous data. Usually, this clustering method creates more OTUs than manual clustering on phylogenetic trees, which explains the slightly lower Good’s average of this study. However, rarefaction curves are similar to those from previous studies [6, 16] (see S2 and S3 Figs).

A total set of 1830 clones of 16S rRNA was sequenced (between 41 and 96 sequences per library: i.e., branchiostegites, scaphognathites, foregut and midgut) to study the bacterial communities associated with *R*. *chacei* specimens from the different sites (Table 1). Taxonomy was assigned for each sequence and summarized in Table 4. Furthermore, based on 97% similarity, sequences were clustered in OTUs (the number of OTUs per taxonomy units is also summarized in Table 4). In our data, 12 groups were identified (all samples combined), listed below according to the number of clones retrieved: *Epsilonproteobacteria*, *Gammaproteobacteria*, Deferribacteres, Mollicutes, *Alphaproteobacteria*, Bacteroidetes, Candidate division OD1, *Deltaproteobacteria*, *Zetaproteobacteria*, *Lentisphaerae*, *Betaproteobacteria* and Firmicutes. Studies based on PCR amplifications and clone libraries are known to underestimate genetic diversity and can be biased [40]. Nevertheless, our results showed that diversity associated with the *R*. *chacei* cephalothorax (i.e., branchiostegite and scaphognathite samples) was mostly represented by *Epsilonproteobacteria-* and *Gammaproteobacteria-*related sequences, while that of the gut (i.e., foregut and midgut samples) was mostly represented by Deferribacteres- and Mollicutes-related sequences (Table 4). For our phylogenetic analysis we therefore focused our study on these four main lineages, using only forward sequences for quality in the phylogenetic reconstruction, and excluding singleton OTUs.

**Table 4:** Clone library results (based on partial 16S rRNA genes sequences).

taxon	Total clone number per lineage	1	2	3	4	5	6	7	8	9	10	11	12	13	14	15	16	17	18	19	20	21	Total OTUs 97% («Forwards» data)
*Epsilonproteobacteria*	890	17	17	2	8	44	4	11	62	87	44	38	90	34	83	36	68	58	28	40	62	57	45
*Gammaproteobacteria*	352	1					74	45	1		34	35	3	1	4	3	4	27	50	41	7	22	23
Deferribacteres	211	23		70	81		13	24															2
Mollicutes (Tenericutes)	187	43	67	19		43	1	12		2													4
*Alphaproteobacteria*	79				2	1					6	6		5		4	20	5	3	2	19	6	8
Bacteroidetes	42	3	1		1			1		2	3	5		1	5			1	8	5	5	1	9
Candidate_division_OD1	31							1	27	1										2			3
BD1-5	20								1	1	1				1		2	3	6	5			7
*Deltaproteobacteria*	9					1				2	3	3											3
*Zetaproteobacteria*	5								1		1						1				2		1
Lentisphaerae	2								2														1
*Betaproteobacteria*	1	1																					1
Firmicutes	1					1																	1
**Total clone number**	1830	88	85	91	92	90	92	94	94	95	92	87	93	41	93	43	95	94	95	95	95	86	

1 = *R*.*chacei*C40Bb-LS-MG, 2 = *R*.*chacei*C40BbLS-FG, 3 = *R*.*chacei*C08BbRB-MG, 4 = *R*.*chacei*C04Bb-RB-MG, 5 = *R*.*chacei*C08Bb-RB-FG, 6 = *R*.*chacei*C07Bc-TAG-MG, 7 = *R*.*chacei*C01Bc-SP-MG, 8 = *R*.*chacei*C09Bc-TAG-FG, 9 = *R*.*chacei*C04Bc-SP-FG, 10 = *R*.*chacei*C40Bb-LS-SC, 11 = *R*.*chacei*C40Bb-LS-LB, 12 = *R*.*chacei*C08Bb-RB-SC, 13 = *R*.*chacei*C01Mom-RB-LB, 14 = *R*.*chacei*C08Bb-RB-LB, 15 = *R*.*chacei*C01Mom-RB-SC, 16 = *R*.*chacei*C04Bb-RB-SC, 17 = *R*.*chacei*C04Bb-RB-LB, 18 = *R*.*chacei*C07Bc-TAG-SC, 19 = *R*.*chacei*C01Bc-SP-LB, 20 = *R*.*chacei*C07Bc-TAG-LB, 21 = *R*.*chacei*C01Bc-SP-SC. Names of librairies are coded as follow: *R*.*chacei* indicates the name of the species; C40 the number of the individual; Bb, Bc, Mom stands for the name of the cruises (ie Bb: BioBaz 2013, Bc: BICOSE 2014, Mom: MoMARdream-Naut 2007); LS, RB, TAG, SP stands for the name of the sampling site (ie LS: Lucky Strike, RB: Rainbow, TAG, SP: Snake Pit); and MG, FG, SC, LB stands for the type of sample (ie MG: mid gut, FG: foregut, SC: scaphognathite, LB: Branchiostegite).

Rarefaction curves (S2 and S3 Figs) indicated that clone libraries did not exhaust all the diversity, but mostly reached a plateau. Good’s coverage confirmed the rarefaction curve observations, with an average of 80.75% (+/- 9.65%), correctly describing bacterial community diversity associated with the shrimp. *Alpha* diversity calculated with Simpson index and Simpson evenness highlighted diversity profiles typical of symbiosis: low diversity with low equitability, reflecting the dominance of some phylotypes. *Alpha* diversity was lower in the gut (1-Simpson < 0.7) than in the cephalothorax (1-Simspon > 0.7). *Beta* diversity based on Bray–Curtis analysis confirmed differences between gut and cephalothorax community composition (S4 Fig).

### 16S rRNA phylogeny among the *Epsilonproteobacteria*-related sequences

**Fig 6:**
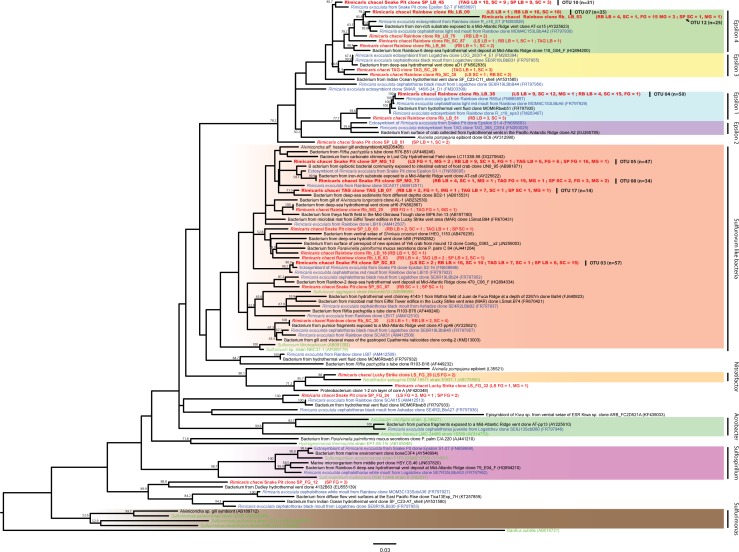
*Epsilonproteobacteria* 16S rRNA gene phylogenetic tree calculated on 624 bp, with the neighbor-joining method, the GTR model [66] and 1000 bootstrap resamplings. Bacterial sequences of *Rimicaris chacei* found in this study are labeled in red, *Rimicaris exoculata* symbionts in blue, and cultivated microbial strains in green. The number of clone sequences per site and per sample are given in brackets (Sites: RB: Rainbow, SP: Snake Pit, LS: Lucky Strike. Samples: LB: branchiostegites, SC: scaphognathites, MG: midgut, FG: foregut).

16S rRNA genes sequences related to the *Epsilonproteobacteria* within *R*. *chacei* cephalothorax clone libraries were distributed among 24 OTUs, clustering within *R*. *exoculata* cephalothoracic [13, 14, 16, 41] and digestive tract clone sequence groups [6–8]. *R*. *chacei* OTUs were mainly distributed within two phylotypes. The first phylotype was related to *Sulfurovum* species, and the second to *Epsilon* groups 1 to 4 according to the nomenclature of Petersen *et al*. [14] (Fig 6). However, *Sulfurovum*-related OTUs found in *R*. *chacei* were also closely related to sequences from hydrothermal vent systems worldwide. The closest cultured bacteria related to the most represented phylogenetic group were *Sulfurovum lithotrophicum* [42], *Sulfurovum aggregans* [43] and *Sulfurovum* sp. strain NBC37-1 [44]. These species are all known to be sulfur-oxidizing chemolithoautotrophic bacteria, isolated from hydrothermal vents. The other phylogenetic groups composed by *Epsilon* 1 to 4 were not represented by cultured strains, but were close to *R*. *exoculata* symbiont clusters [14].

### 16SrRNA phylogeny of the *Gammaproteobacteria*-related sequences

**Fig 7:**
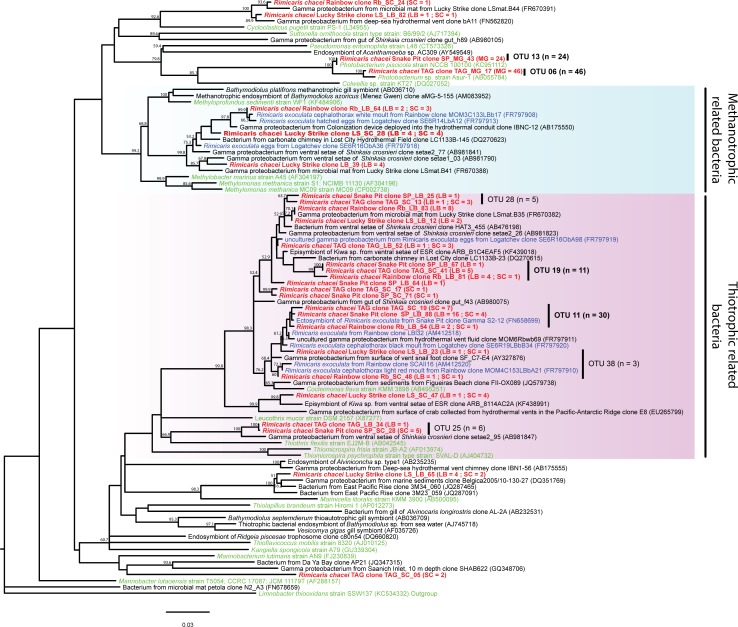
*Gammaproteobacteria* 16S rRNA gene phylogenetic tree calculated on 685 bp, with the neighbor-joining method, the GTR model [66] and 1000 bootstrap resamplings. Bacterial sequences associated with *Rimicaris chacei* are labeled in red, *Rimicaris exoculata* symbionts in blue, and cultivated microbial strains in green. The number of clone sequences per vent site and per sample is given in brackets (abbreviations for sites and samples are as in Fig 6).

16S rRNA genes sequences of *R*. *chacei* clone libraries affiliated to *Gammaproteobacteria* clustered with previously described *R*. *exoculata* cephalothoracic [13, 14, 16, 41] and digestive tract clones [6–8]. Two main phylogenetic groups were identified (Fig 7). The first one was closely related to *Methyloprofundus sedimenti* [45], *Methylobacter marinus* [46], and *Methylomonas methanica* [47]. These cultivated bacteria are known to be methanotrophic. The second one was closely related to *Thiothrix flexilis* [48–50], *Thiomicrospira frisia* [51], *Thiomicrospira psychrophila* [52] and *Cocleimonas flava* [53], all known to be chemoautotrophic sulfur-oxidizers. Five OTUs were not related to these phylogenetic groups and were related to methanotrophic or thiotrophic phylogenetic groups, close to environmental sequences obtained from hydrothermal vents. TAG_SC_05, LS_LB_82 and LS_LB_65 clones represent OTUs with 2, 3 and 7 sequences in clone libraries, respectively, and were close to environmental sequences. The two most highly represented OTUs in our clone libraries (i.e., SP_MG_43 and TAG MG_17 clones) were only found in the midgut of *R*. *chacei* from Snake Pit and TAG, respectively, and did not cluster with any known sequences related to hydrothermal vents or symbiosis.

### 16S rRNA phylogeny of the Deferribacteres-related sequences

**Fig 8:**
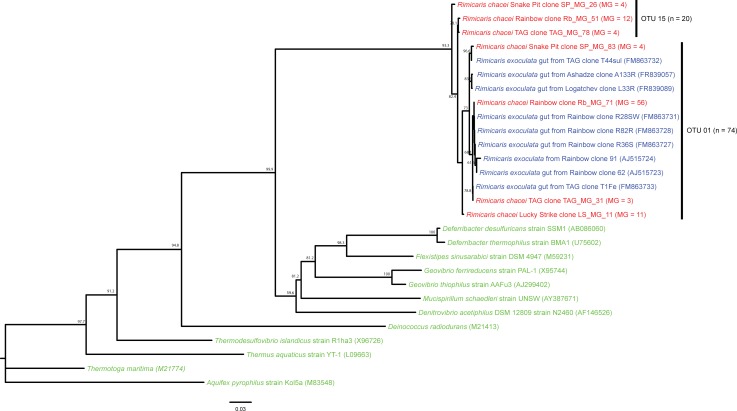
Deferribacteres 16S rRNA gene phylogenetic tree calculated on 764 bp, with the neighbor-joining method, the GTR model [64] and 1000 bootstrap resamplings. Bacterial sequences of *Rimicaris chacei* found in this study are labeled in red, *Rimicaris exoculata* symbionts in blue, and cultivated microbial strains in green. The number of clone sequences per vent site and per sample is given in brackets (abbreviations for sites and samples are as in Fig 6).

Compared to *Epsilon* and *Gammaproteobacteria-*related OTUs, the diversity of Deferribacteres-related sequences was very low, even though this taxonomic group was found in apparently high abundance in our libraries. Only two OTUs were identified exclusively in the four midgut samples (Fig 8). The OTU represented by the clone Rb_MG_71, was present at the hydrothermal vent sites, whereas the other, represented by the clone TAG_MG_78, was absent from Lucky Strike. These 2 OTUs were closely related to previously known *R*. *exoculata* gut symbionts [6]. The closest related cultured bacteria to this phylogenetic group were *Deferribacter thermophilus* [54] and *Deferribacter desulfuricans* [55], both known to be heterotrophic thermophilic and anaerobic metal-reducers, especially of iron or sulfur.

### 16S rRNA phylogeny of the Mollicutes-related sequences

**Fig 9:**
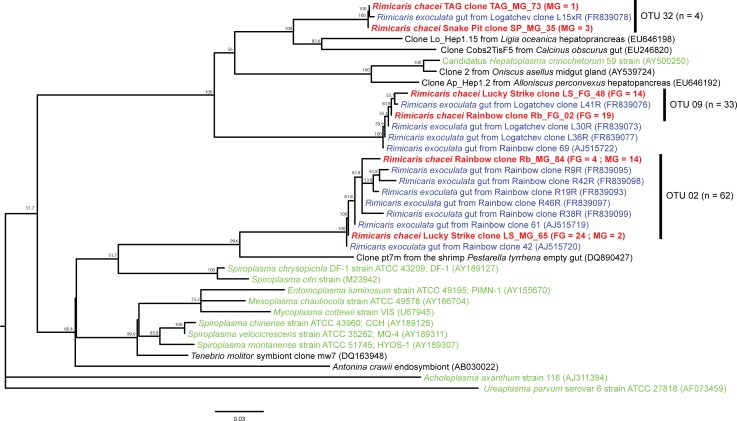
Mollicutes 16S rRNA gene phylogenetic tree calculated on 657 bp, with the neighbor-joining method, the GTR model [64] and 1000 bootstrap resamplings. Bacterial sequences of *Rimicaris chacei* found in this study are labeled in red, *Rimicaris exoculata* symbionts in blue, and cultivated microbial strains in green. The number of clone sequences per vent site and per sample is given in brackets (abbreviations for sites and samples are as in Fig 6).

As for the Deferribacteres, the diversity of Mollicutes-related clones was lower than that of *Epsilon* and *Gammaproteobacteria* ones, but still well represented in our libraries obtained from stomach and midgut samples. Three main OTUs (and one singleton) were identified (Fig 9) and were closely related to *R*. *exoculata* gut symbionts, but still not to environmental sequences. The OTU represented by clone Rb_FG_02 was present in the foregut of Rainbow and Lucky Strike shrimp, and in the midgut of Rainbow shrimp. The OTU represented by the clone SP_MG_35 was present in the foregut of Rainbow and Lucky Strike shrimp. The OTU represented by the clone LS_MG_65 was only present in TAG and Snake Pit midgut samples.

### Fluorescent *in situ* hybridization

We used FISH microscopy to merge microscopic observations with molecular analysis as far as possible. Development and tests were done to determine optimal hybridization temperature (here 46°C) and stringency (here 40% formamide), and to check specific hybridization for each probe listed in Table 2. Only Eub338 (universal bacterial probe), Epsy 549 (specific to *Epsilonproteobacteria*) and Gam42a (specific to *Gammaproteobacteria*) presented clear hybridization results. Several trials were carried out using archaeal probes, but were unsuccessful.

**Fig 10:**
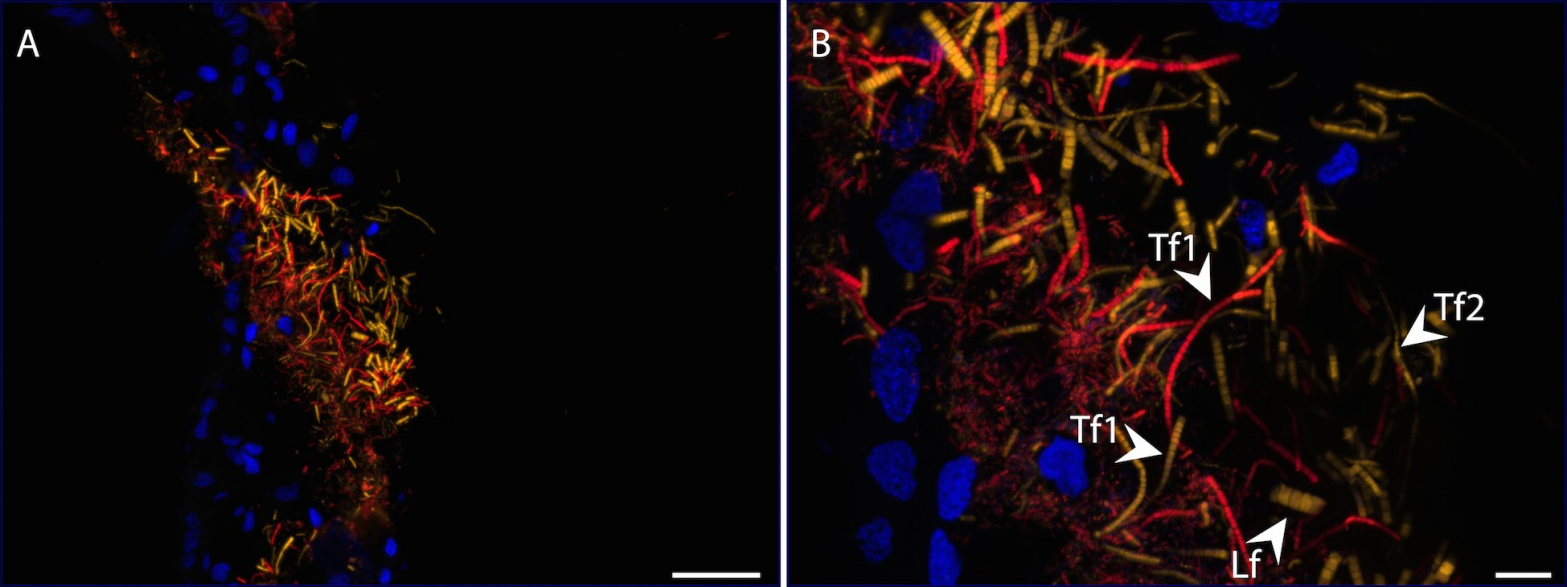
FISH observations of a branchiostegite from a specimen from Snake Pit site. A) Overview of a branchiostegite stained with DAPI (blue) and hybridized with *Epsilonproteobacteria* general probe Epsy549-Cy3 (yellow) and *Gammaproteobacteria* general probe Gam42-Cy5 (red). B) Higher magnification of the different morphotypes. Arrows point to filamentous morphotypes described previously (Lf: large filaments, Tf1: thin filaments of type 1 and Tf2: thin filament of type 2). Scale bars: B = 50 μm, C = 10 μm.

On branchiostegites and scaphognathites, *Epsilonproteobacteria* and *Gammaproteobacteria* were the most abundant hybridized cells. Both lineages were represented by filamentous morphotypes. *Epsilonproteobacteria* were represented by large filaments and thin filaments of types 1 and 2 (in yellow on Fig 10A and 10B). *Gammaproteobacteria* were represented by thin filaments of type 1 (in red on Fig 10A and 10B). Some rods hybridized with the Gam42a probe, but the resolution of the image does not allow us to confidently associate them with one of the two types described above. Methanotrophic-like bacteria located in and on the mineral crust failed to give any conclusive fluorescent signal.

In the midgut, none of the probes tested hybridized to bacteria, except the general bacterial probe Eub338, as already observed for *R*. *exoculata* [6, 8]. Furthermore, shrimp epithelial cells were autofluorescent by Cy3 emission. Using this apparent bias, we were able to observe that bacteria were present at high density at the apex of the midgut epithelial cells (Fig 5B).

## Discussion

### Similarities between symbiotic communities of *R*. *chacei* and *R*. *exoculata*

Our study confirms the first observations [10, 11] and deepens our knowledge on the localization and morphology of the symbionts of *R*. *chacei*, and highlights strong similarities with *R*. *exoculata* symbiosis.

Firstly, although the bacteriophore tissues are less developed in *R*. *chacei* than in *R*. *exoculata* (i.e., branchiostegites and scaphognathites are not hypertrophied, bacteriophore seta on the scaphognathites are less numerous, areas covered by bacteria on the inner face of the branchiostegites are smaller), the cephalothoracic community of *R*. *chacei* also forms a dense mat on the anterior third of the inner side of the branchiostegites and on both sides of the scaphognathites (Fig 1A and 1B, [12]). In both species, epibionts of the cephalothorax are associated with iron oxide and sulfide deposits (Figs 1A, 1B, 3F and 3G, [12, 56]).

Secondly, associated bacteria of the two species are morphologically similar. Using SEM observations, Casanova *et al*. [10] described three morphotypes (rods, thin and thick filaments) among the cephalothoracic epibionts of *R*. *chacei*, similar morphotypes were also described in *R*. *exoculata* by these authors, with bacteria of roughly similar sizes. Combining SEM and TEM observations allowed us to refine this first description and revealed a larger diversity, with six bacterial morphotypes (see Table 3 for measures): i) two rod types: a long thin and a short thick type (Fig 2E); ii) 3 types of filaments including 2 types of thin filaments, differing in the size and shape of their cells (Figs 2D, 3A and 3C), and iii) a coccobacillus morphotype, corresponding to methanotrophic-like bacteria, never observed before in *R*. *chacei*. These six morphotypes correspond to those described for *R*. *exoculata* using the same combined approach of SEM and TEM [13].

In the digestive tract, only one morphotype was observed in all samples analyzed, located in the mid-gut, inserted between the microvilli of the epithelial gut cells and separated from the bolus by the peritrophic membrane. These bacteria are long thin filaments made of individual cells without any visible septum. Similar bacteria were described in the *R*. *exoculata* midgut by Durand *et al*. [6]. Observations of bacteria contained in the bolus are difficult due to the high mineral load. Some rods and cocci were nevertheless observed (not shown), most of which were relatively undamaged, as already observed in *R*. *exoculata* [7].

Thirdly, associated bacteria of the two species are closely phylogenetically related. Phylogenetic studies on *R*. *exoculata* cephalothoracic communities revealed a large phylogenetic diversity with two major groups: *Epsilonproteobacteria* and *Gammaproteobacteria*, related to chemoautotrophic bacteria [13, 14, 16, 17]. Phylogenetic analyses on *R*. *exoculata* gut symbionts have led to the identification of four major clades: Mollicutes, Deferribacteres, *Gammaproteobacteria* and *Epsilonproteobacteria* [6–8, 57]. The same four phylotypes are well represented in our *R*. *chacei* clone libraries, with *Epsilonproteobacteria* and *Gammaproteobacteria* more abundant in the cephalothorax, as seen on FISH images, and Deferribacteres and Mollicutes only present in the gut.

In this study, some methanotrophic-like bacteria were observed at Rainbow and Lucky Strike, but not at Snake Pit, nor TAG. In 2012, Guri et al. [16] also found methanotrophic-like bacteria on adult *R*. *exoculata* from the Rainbow site. Although less abundant, this lineage may be of importance in the symbiosis of the shrimp and is probably methane dependent [16].

### Potential trophic role of symbionts and mixotrophy of *R*. *chacei*

The similarities, in terms of location, morphology and phylogeny of the symbiotic communities of these two *Rimicari* species could suggest similarities in their diets. Some morphological characters should be taken into account when considering the potential diet of *R*. *chacei*, compared with *R*. *exoculata*: 1) the stomach volume is almost twice as large in *R*. *chacei* as in *R*. *exoculata* [10], implying a greater digestive function. 2) The mandibles are reduced and not used for feeding in *R*. *exoculata*, but show normal development in *R*. *chacei* and are functional [10]. 3) The two pairs of chelipeds are relatively small and cannot extend out of the branchial chamber in *R*. *exoculata* (making food collection out of cephalothorax difficult), which again contrasts with *R*. *chacei* [10]. Taken together, these data could suggest a mixotrophic diet in *R*. *chacei*, with a classic process of collection, crushing, ingestion and digestion, as indicated by the presence of organic material in the gut (observed in this study and also reported by Casanova *et al*. [10] and Segonzac *et al*. [11]). The occurrence of bacterial communities in the *R*. *chacei* cephalothorax, which are even less abundant than in *R*. *exoculata*, suggest that the *R*. *chacei* diet could be supplemented by an input of organic carbon from the chemoautotrophic epibiotic bacteria. Carbon stable isotopes indeed show that *R*. *chacei* has an intermediate δ^13^C signature (-16.1 to -12.3 ‰, [58]) between *R*. *exoculata*, which relies mostly on its epibionts for its diet [18], and *Mirocaris fortunata*, which is opportunist and feeds upon mussels, shrimp or other invertebrates when available [26]. Transcuticular transfer of bacterial byproducts to shrimp was demonstrated in *R*. *exoculata* [9] using *in vivo* incubations with isotope-labeled inorganic carbon. The hypothesis of this mode of nutrition arose from several observations: i) the absence of areas scraped by the animal, or free of bacteria on the inner side of branchiostegites or on scaphognathites [12, 27], refuting the long-argued hypothesis that shrimp feed on its cephalothoracic symbionts, scraping them on the inner face of the branchiostagite with its first 2 pairs of pereiopods enclosed within the carapace [18, 20, 59], ii) the thinness of the branchiostegite inner cuticle lining in contact with the bacteria (between 0.5 μm and 5 μm). Regarding *R*. *chacei*, no scraped areas on the inner side of branchiostegites were ever observed on the samples studied. Furthermore, the thickness of the branchiostegite inner cuticle is of the same order of magnitude as in *R*. *exoculata* (5.1 ± 0.9 μm for *R*. *exoculata*, n = 16 and 5.5 ± 2.2 μm for *R*. *chacei*, n = 16, data not shown). These morphological features could suggest that a transcuticular transfer of small organic molecules of bacterial origin also occurs in *R*. *chacei*. *In vivo* experiments with isotope-labeled inorganic carbon should be carried out to obtain firm evidence of a transfer of organic material from epibionts to their host and a real trophic symbiosis between *R*. *chacei* and its epibionts.

The conservation of a mixed diet with two potential sources of food would allow switching between food sources in the case where there is a shortage of one type. Species with a mixotrophic diet based in part on the organic molecules supplied by symbiotic bacteria, combined with an external contribution have already been described in hydrothermal symbiotic species such as bathymodiolin mussels or some galatheid crabs [60, 61]. For example, *Bathymodiolus azoricus* obtains energy from both a dual endosymbiosis and filter-feeding [62]. Martins *et al*. [60] observed a spatial segregation for *B*. *azoricus*, with the largest specimens (which strongly rely on their symbionts) living in the warmest areas where there is a higher concentration of reduced compounds required by bacteria, and the smaller individuals (which depend strongly on filter-feeding, [60]) found further away from the vent flow. Such segregation is also observed for the two *Rimicaris* species: *R*. *exoculata*, which obtains most of its energy from its epibionts, lives close to the vent emission, while *R*. *chacei*, which is presumably mixotrophic and depends only partly on its symbionts, lives further away from fluid outlets [11], where chemoautotrophic production is lower.

### Does mixotrophy in *R*. *chacei* result from competition?

*R*. *chacei* and *R*. *exoculata* live on the same MAR vent sites, on overlapping habitats, the former being restricted to the areas surrounding the aggregates of the latter [11, 24]. *R*. *exoculata* is always the most abundant species in these habitats [10], suggesting that its fully symbiotic diet is able to sustain a greater biomass than the mixotrophic strategy of *R*. *chacei*. We can speculate that this situation is due to competition with *R*. *exoculata*, that would maintain *R*. *chacei* at a distance from the nourishing fluid, and thus in a less effective symbiosis, requiring complementary contributions of a mixotrophic diet. Interestingly, recent phylogenetic analyses [21, 63, 64] showed that the closest known relative to *R*. *chacei* is the Caribbean species *R*. *hybisae*. Teixeira *et al*. [62] suggested that they could even be a single species. *R*. *hybisae* lives in the Mid-Cayman Trough on the western Caribbean Sea and presents the same characteristic enlarged cephalothorax as *R*. *exoculata* [22]. Isotopic data showed that it predominantly relies on its cephalothoracic ectosymbionts for its organic carbon needs [65]. Despite their strong phylogenetic proximity, *R*. *chacei* and *R*. *hybisae* exhibit morphological and nutritional differences related to the extent of their symbiotic development. This suggests that *R*. *chacei* maintains a mixed strategy due to niche competition with *R*. *exoculata*, in contrast with *R*. *hybisae* that has full access to vent fluid to fuel its symbiotic bacteria, lacking any known competitor on the Mid-Cayman Trough.

In conclusion, these results suggest that the presence of bacterial symbiosis in these vent shrimps could be considered as an adaptive mechanism leading to the dominance of one species when occurring on the vent site but also as an evolutive driver in the context of the species colonization on MAR. These hypotheses have to be tested further by phylogenetic approaches.

## Supporting information

S1 Fig Stereomicroscopic view of *R*. *chacei* stomachs and midguts. (A) Stomach of an individual from Rainbow; (B) midgut of an individual from TAG; stomach (C) and midgut (D) of an individual from Snake Pit; and stomach (E) and midgut (F) of an individual from Lucky Strike sites. Scale bars: A, C, E = 1 mm; B, D, F = 200 μm.
(JPG)

S2 Fig Rarefaction curves of cephalothoracic clone libraries of *R*. *chacei* samples. (DOCX)

S3 Fig Rarefaction curves of digestive tract clone libraries of *R*. *chacei* samples. (DOCX)

S4 Fig Dendogram of all analyzed samples, based on Bray–Curtis beta diversity on the left, and taxonomy of sequences associated with each sample on the right. (DOCX)
