# Supplementary figures and images for: Gill chamber and gut microbial communities of the hydrothermal shrimp Rimicaris chacei Williams and Rona 1986: A possible symbiosis
Source: PLoS One. 2018 Nov 2;13(11):e0206084. doi: 10.1371/journal.pone.0206084 (PMC6214521; doi:10.1371/journal.pone.0206084)

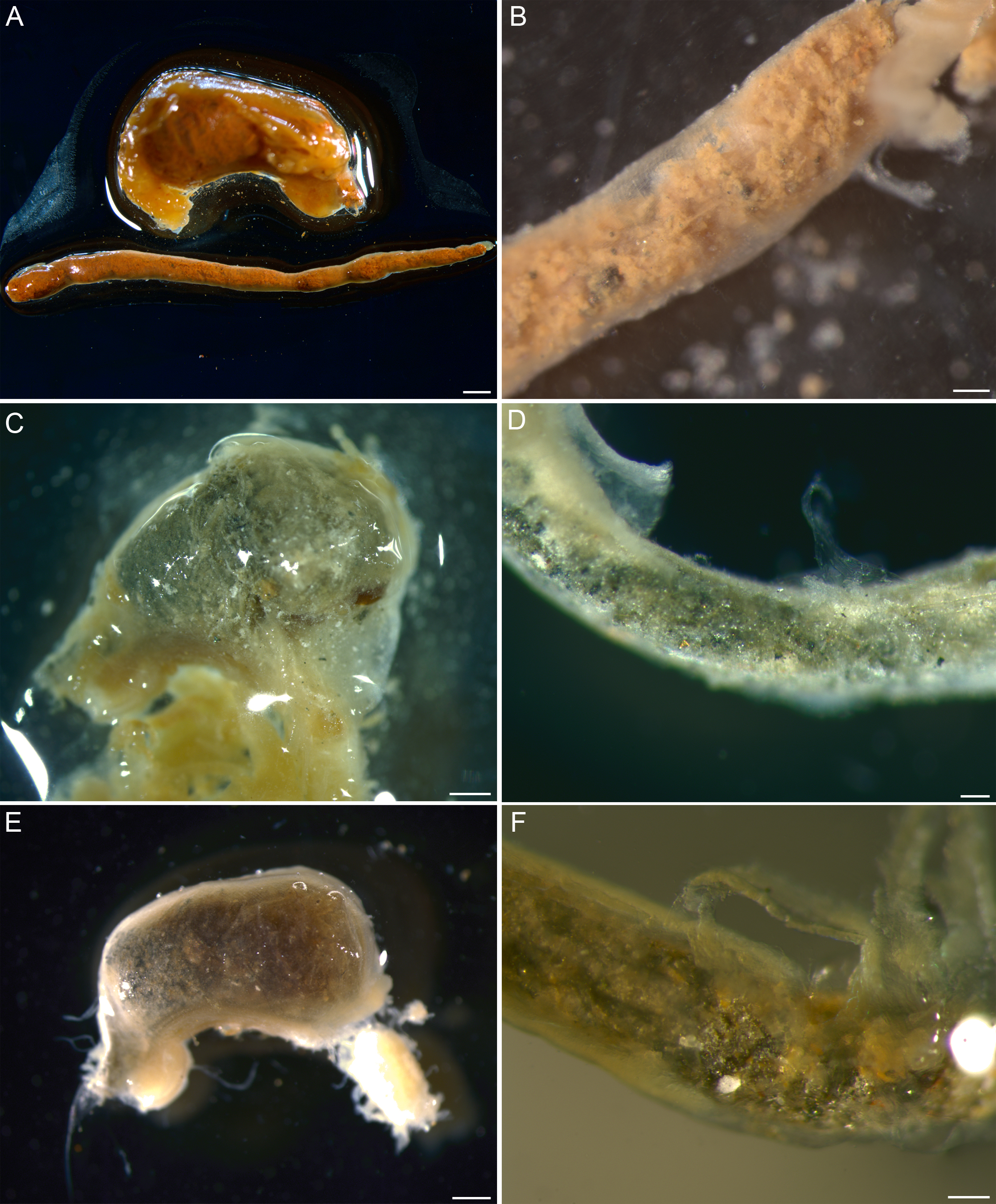

Supplement: S1 Fig — (A) Stomach of an individual from Rainbow; (B) midgut of an individual from TAG; stomach (C) and midgut (D) of an individual from Snake Pit; and stomach (E) and midgut (F) of an individual from Lucky Strike sites. Scale bars: A, C, E = 1 mm; B, D, F = 200 μm. (JPG) [file pone.0206084.s001.jpg]
