# Supplementary material for: Gill chamber and gut microbial communities of the hydrothermal shrimp Rimicaris chacei Williams and Rona 1986: A possible symbiosis
Source: PLoS One. 2018 Nov 2;13(11):e0206084. doi: 10.1371/journal.pone.0206084 (PMC6214521; doi:10.1371/journal.pone.0206084)

Figure S4 : Dendogramme of all analysed samples based on Bray-Curtis beta diversity on left, and taxonomy of each sequences associated to each samples on right.


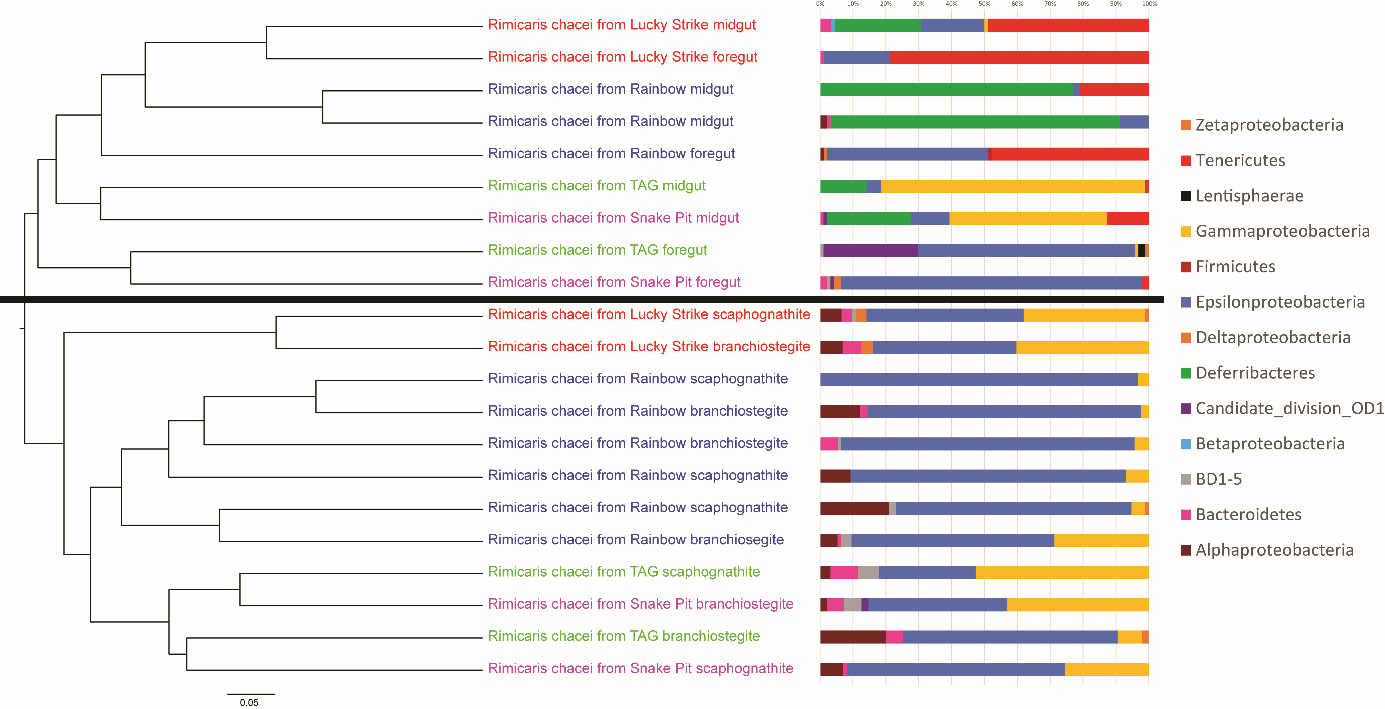

Supplement: S4 Fig — (DOCX) [file pone.0206084.s004.docx]
